# Hyperparameter Tuning and Automatic Image Augmentation for Deep Learning-Based Angle Classification on Intraoral Photographs—A Retrospective Study

**DOI:** 10.3390/diagnostics12071526

**Published:** 2022-06-23

**Authors:** José Eduardo Cejudo Grano de Oro, Petra Julia Koch, Joachim Krois, Anselmo Garcia Cantu Ros, Jay Patel, Hendrik Meyer-Lueckel, Falk Schwendicke

**Affiliations:** 1 Department of Oral Diagnostics, Digital Health and Health Services Research, Charité Center for Oral Health Sciences CC3, Charité–Universitätsmedizin Berlin (Corporate Member of Freie Universität Berlin and Humboldt-Universität zu Berlin), Aßmannshauser Straße 4-6, 14197 Berlin, Germany; jose-eduardo.cejudo@charite.de (J.E.C.G.d.O.); joachim.krois@charite.de (J.K.); anselmogarciacantu@gmail.com (A.G.C.R.); 2 Department of Orthodontics and Dentofacial Orthopedics, Charité Center for Oral Health Sciences CC3, Charité–Universitätsmedizin Berlin (Corporate Member of Freie Universität Berlin and Humboldt-Universität zu Berlin), Aßmannshauser Straße 4-6, 14197 Berlin, Germany; petra-julia.koch@charite.de; 3 Health Informatics, Department of Health Services Administrations and Policy, Temple University College of Public Health, Philadelphia, PA 19122, USA; patel.jay@temple.edu; 4 Department of Restorative Preventive and Pediatric Dentistry, zmk Bern, University of Bern, 3012 Bern, Switzerland; hendrik.meyer-lueckel@zmk.unibe.ch

**Keywords:** artificial intelligence, deep learning, modeling, orthodontics, photographs

## Abstract

We aimed to assess the effects of hyperparameter tuning and automatic image augmentation for deep learning-based classification of orthodontic photographs along the Angle classes. Our dataset consisted of 605 images of Angle class I, 1038 images of class II, and 408 images of class III. We trained ResNet architectures for classification of different combinations of learning rate and batch size. For the best combination, we compared the performance of models trained with and without automatic augmentation using 10-fold cross-validation. We used GradCAM to increase explainability, which can provide heat maps containing the salient areas relevant for the classification. The best combination of hyperparameters yielded a model with an accuracy of 0.63–0.64, F1-score 0.61–0.62, sensitivity 0.59–0.65, and specificity 0.80–0.81. For all metrics, it was apparent that there was an ideal corridor of batch size and learning rate combinations; smaller learning rates were associated with higher classification performance. Overall, the performance was highest for learning rates of around 1–3 × 10^−6^ and a batch size of eight, respectively. Additional automatic augmentation improved all metrics by 5–10% for all metrics. Misclassifications were most common between Angle classes I and II. GradCAM showed that the models employed features relevant for human classification, too. The choice of hyperparameters drastically affected the performance of deep learning models in orthodontics, and automatic image augmentation resulted in further improvements. Our models managed to classify the dental sagittal occlusion along Angle classes based on digital intraoral photos.

## 1. Introduction

Deep learning (DL) has been employed for image analysis (“computer vision”) in a range of medical fields; in dentistry, DL is increasingly established for identifying pathologies such as caries or apical lesions, periodontal bone loss, or intra-bony defects on imagery; see [1,2] for recent reviews. A major field of activity is orthodontics, specifically landmark detection on cephalometric radiographs [3] and, recently, the determination of growth and development periods [4]. In many circumstances, DL shows accuracies similar or even superior to those of experts [5,6] while increasing the efficiency and reliability of any analyses.

In non-medical domains, DL usually involves millions of data points (images) and labels (annotations) to allow for learning the structure in the data representing the labels. Such large, labelled datasets are absent in medicine and more so in dentistry, as obtaining vast amounts of data points, specifically images, is challenging due to data protection regulations. Moreover, labeling images is time-consuming, expensive, and error-prone. Hence, it is relevant to also leverage smaller labelled datasets. Among others, two strategies allow to improve the accuracy of DL models for image analysis (also called computer vision) trained on smaller datasets: hyperparameter tuning and image augmentation.

A DL model has multiple hyperparameters, and two prominent examples are the batch size, which is the number of datapoints to train the system in a single pass, and the initial learning rate, which determines the step size taken by the optimizer and therefore how fast the model learns. When training DL models on small datasets, in many cases, the choice of hyperparameters has a significant effect on the performance of the models [7]. At the same time, hyperparameter tuning is a challenging problem since the number of combinations grows exponentially with the number of hyperparameters. Given this combinatorial complexity, a brute-force hyperparameter search (grid search) often becomes computationally intractable due to the large number of possible combinations and to the cost of having to train one or several models for each of them for the purpose of comparisons. Consequently, random hyperparameter search is applied most frequently and has proven to be effective and more efficient than grid-search for DL [8]. Hyperparameter optimization is present in medical imaging research, where it has been used to obtain models with higher diagnostic performance for a range of problems [9,10].

Image augmentation is another technique used in computer vision for improving the performance of models trained on small datasets. During augmentation, copies of images are created by a sequence of transformations (flipping, rotations, color transformation, etc.). Image augmentation is known to improve the performance and robustness of computer vision models and can be used to induce invariances and symmetries without having to modify the model’s architecture. Intuitively, it can also be interpreted as a way of creating extra images to interpolate gaps in the data manifold. In this manner, the model is trained with a dataset containing richer features than a dataset without augmentation. Notably, though, designing a good image augmentation pipeline for a particular problem requires expert knowledge. Moreover, the components of the augmentation policy might have both continuous and discrete parameters, such as the angle of rotation or the kernel size for blurring, apart from the probability of every component being applied. Finding a good augmentation policy can be formulated as a search problem. However, searching in this space is computationally expensive, too. Therefore, in summary, there is a gap in knowledge whether the hyperparameter tuning or automatic image augmentation would enhance the classification performance especially while utilizing small, manually labelled datasets. Image augmentation techniques have appeared in the medical imaging literature, playing a particularly relevant role in self-supervised learning [11]. Automatic image augmentation has been used for improving the performance of models [12,13,14]. As per our best knowledge, no study in dentistry has attempted to determine the accuracy performance of the hyperparameter tuning or automatic image augmentation on dental images.

**Figure 1:**
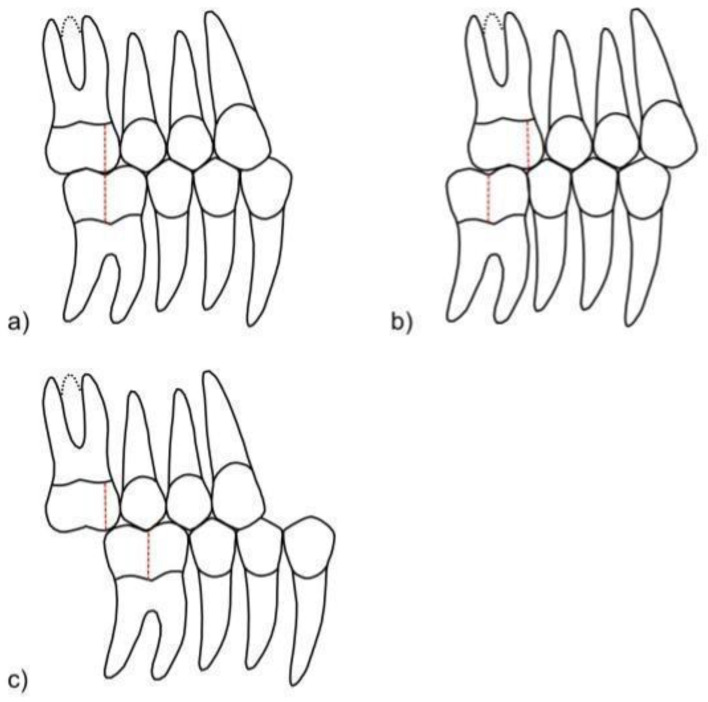
Schematic representation of different Angle classes: (**a**) Angle class I, (**b**) Angle class II, and (**c**) Angle class III.

As a result, in the present study, we examined the potential of automatic hyperparameters and image augmentation search techniques for computer vision problems in images related to orthodontics. We formulated an image classification task for photographs taken for diagnostic reasons before the start of orthodontic treatment, with each photo being classified as Angle classes I, II, and III according to the sagittal relationship of the upper and lower first molars [15]. In orthodontics, the Angle classification is a basic concept for describing the sagittal occlusal relationship between the upper and lower first molars on each side for the permanent dentition. By Angle’s definition, a normal (neutral) occlusion (Angle class I) is given when the mesialbuccal cusp of the upper first molar occludes in the groove between the mesial and the distal (or, if present, middle) buccal cusps of the lower first molar [16], and the rest of the teeth in the arch are aligned accordingly [17]. Ever since and despite its controverse inadequateness [17,18,19,20,21], the Angle classification is a routine diagnostic assessment in orthodontics [22] that distinguishes between a neutral sagittal occlusion and a mesial and distal malocclusion that could lead to masticatory limitations and unphysiological tooth wear [23] (Figure 1) and could indicate an orthodontic treatment.

We hypothesized that using hyperparameter tuning and automatic image augmentation could significantly improve the classification performance of a deep learning classifier of photos along different Angle classes.

## 2. Materials and Methods

### 2.1. Study Design

In the present retrospective cohort study, we used a dataset of photographs classified into different Angle classes to assess hyperparameter tuning using the asynchronous successive halving algorithm (ASHA) algorithm [24] and automated image augmentation policy search using faster autoaugment [25]. Using the best hyperparameter combination resulting from the ASHA algorithm, we trained an augmentation policy using faster autoaugment. We then compared a model trained with the resulting policy with a model tuned and trained without augmentations. We used the explainable AI technique GradCAM to interpret the predictions of the model. Reporting of this study follows the Standards for Reporting of Diagnostic Accuracy Studies (STARD) guidelines [26], the Checklist for Artificial Intelligence in Medical Imaging (CLAIM) [27], and the Checklist for Artificial Intelligence in Dental Research [28].

### 2.2. Data, Sampling, and Gold-Standard Dataset Development

Our dataset included 2051 clinical RGB images retrieved from the planning and simulation software OnyxCeph (Image Instruments, Chemnitz, Germany) at the Department of Orthodontics and Dentofacial Orthopedics at Charité-Universitätsmedizin Berlin, which were previously taken by the attendings and postgraduate students of the department with the same camera and settings after having received an instruction on how to take intraoral photos in the orthodontic practice. There were 42.1% male and 57.9% female patients; the mean (SD, min-max) age was 18.9 (10.6, 4–60) years. All intraoral photos were taken indirectly through a mirror with a digital CANON 80D reflex camera (Ota, Japan) and a CANON macro lens (focal length 1/200, aperture 22), showing the occlusal relationship between the upper and lower dentition on the right and left side of the mouth. The collection of data was ethically approved (EA4/080/18). One orthodontist (P.J.K.) reviewed the images and classified them into Angle class I (605 photos, 30%), Angle class II (1038, 50%), and Angle class III (408, 20%), respectively. The manually labelled dataset was sporadically checked by one other expert, and disagreements were resolved through consensus and adjudications. The adjudicated dataset was used as a gold-standard dataset to train, test, and validate our computer vision algorithms. 

### 2.3. Data Preparation, Model, and Training

For all our experiments, we used a deep learning ResNet-18 architecture pretrained on the ImageNet dataset as a feature extraction module. We added a classification head with three output neurons equal to the number of categories, followed by a SoftMax activation function. The input of the classification model was an RGB image, and the output a probability distribution over the three Angle classes, the values being interpreted as a confidence score. Angle classes II and III (36% and 20% of the total dataset, respectively) were underrepresented, so we considered the dataset as imbalanced. To address this class imbalance, we used a weighted cross entropy loss function with weights inversely proportional to the frequency of each category. In this way, the model was penalized when it misclassified an underrepresented category. The images were resized into 256 × 256 × 3 tensors and normalized with the mean and standard deviation of the ImageNet dataset. We trained our models on a NVIDIA Quadro RTX 6000 graphics card (NVIDIA, Santa Clara, CA, USA) using the deep learning framework Pytorch.

#### 2.3.1. Hyperparameter Tuning

For this stage of our study, we divided the dataset into a training, validation, and test split, and the performance on the validation set was monitored during training. The splits were stratified, meaning that the original distribution of classes in the splits was the same as the distribution of the entire dataset. In this way, we prevented a low number of underrepresented classes in the test and validation splits. For simplicity, we focused on the batch size and learning rate for hyperparameter tuning since these are two of the parameters that impact the performance of most models [7]. We considered a continuous interval for the learning rate from 10^−6^ to 10^−2^ and values for batch size contained in (8, 16, 32, 64). We randomly sampled 50 combinations of these two parameters, and we trained a model for each of these, using the validation dataset to monitor the performance during training for early stopping. Due to the high computational cost of cross-validation for hyperparameter tuning, we employed a single train, validation, and test split. We monitored the performance of the model on the validation set, applying early stopping after a patience of five epochs. No image augmentation was used at this stage.

We employed an open-source implementation of the ASHA algorithm [24], which is suitable for large-scale parallel computing and makes use of early stopping to avoid unnecessary computations. We evaluated the resulting models on the test dataset and computed the classification metrics accuracy, sensitivity, specificity, and F1-score. We used the ray tune implementation [29].

#### 2.3.2. Automatic Augmentation

The goal of the faster autoaugment algorithm [25] is to automatically obtain an ideal augmentation policy. This problem is formulated using a generative adversarial network with a trainable policy as the generator and a discriminator, which is trained to detect whether an image has been transformed by the policy. This process makes the generator produce images that are close to the original data and are supposed to fill gaps in the data distribution.

Training a model using the resulting policy could result in a better classifier. We used the implementation from the Albumentations library [30], training for 25 epochs with batch size 8 and learning rate 10^−5^ based on the result from hyperparameter tuning. We considered augmentations such as horizontal and vertical flipping and shifting, rotations, cropping, dropout, and color transformations. It is worth mentioning that the most salient feature required for identifying the different Angle classes is the relative position of the upper and lower first molars in the permanent dentition, as previously described. Thus, introducing vertical flipping as an augmentation might change the features of a certain image and could transform it into a different Angle class. We assumed that the resulting augmentation pipeline would avoid transformations that would result in misclassifications.

Once the augmentation policy was trained, we compared the performance of a model trained with this policy with a model without augmentations. We used stratified 10-fold cross-validation with 10 non-overlapping train, validation, and test splits. The distribution across classes in each of the splits was the same as for the entire dataset. For each split, we trained the model for a maximum of 50 epochs, monitoring the performance on the validation set and using early stopping with a patience parameter of five epochs to avoid overfitting. We used a batch size of 8 images and the Adam optimizer with a learning rate of 10^−5^. We monitored the validation loss during training and applied early stopping. We calculated the average value and the 95% confidence interval (95% CI) for the metrics mentioned above across the test splits and independent two-sided *t*-tests with *p* < 0.05 for each of the models. The remaining settings remained as described.

For the comparison between the model trained with faster autoaugment and a baseline without augmentations, we computed the confusion matrix for each test split from cross-validation and averaged them. We also visualized the receiver operating characteristic (ROC) curves and computed the area under the curve (AUC).

### 2.4. Evaluation and Explainaibility

Explainable AI (XAI) is a field of artificial intelligence that seeks to interpret the behavior of machine learning models. XAI techniques allow practitioners to understand the decisions taken by the models. In computer vision, these techniques usually provide saliency maps that highlight the relevant areas of an image relevant for a certain output. We used the GradCAM algorithm [31], which calculates a weighted average of the activation maps of our model.

## 3. Results

We found statistically significant improvement in the classification performance after hyperparameter tuning and image augmentation. Below, we provide detailed classification performances and comparisons.

### 3.1. Hyperparameter Tuning

**Figure 2:**
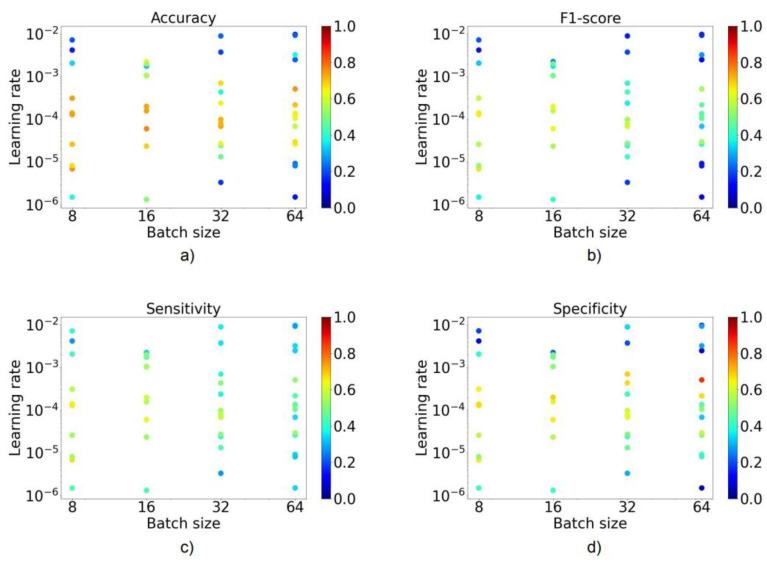
The impact of learning rate and batch size on accuracy in (**a**), F1-score in (**b**), sensitivity in (**c**), and specificity in (**d**). Each dot corresponds to an experiment. Red indicates a value closer to one and blue closer to zero.

Figure 2 contains a visualization of the impact of hyperparameter tuning, i.e., learning rate and batch size, on our accuracy estimates. For all metrics, it was apparent that there was an ideal corridor of batch size and learning rate combinations; generally, smaller learning rates were associated with higher classification performance. The higher the batch size, the smaller the learning rate needed to be to compensate to some degree for lost performance. The combined effect of both factors was significant: performances varied up to 70% between the best and the worst combination. Overall, the performance was highest for learning rates of around 1–3 × 10^−6^ and a batch size of 8 (accuracy 0.63–0.64, F1-score 0.61–0.62, sensitivity 0.59–0.65, and specificity 0.80–0.81), respectively.

### 3.2. Augmentation

**Figure 3:**
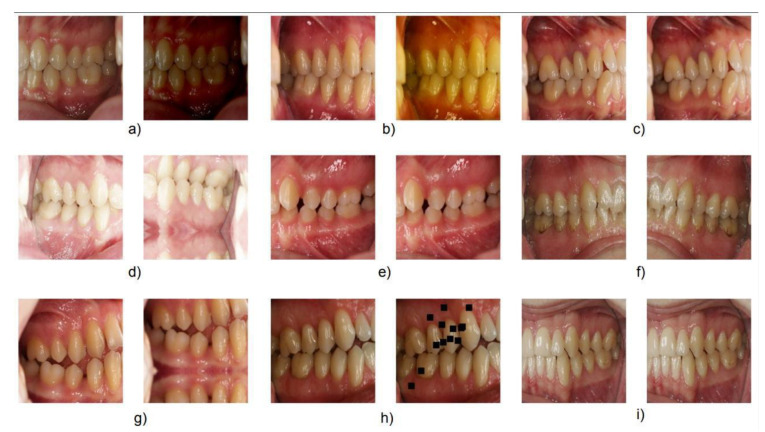
Original images together with their augmented version. We observed color changes in (**a**,**b**), no transformation in (**c**), horizontal and vertical flipping together with vertical shift in (**d**), blurring in (**e**,**i**), horizontal flipping in (**f**), vertical shift in (**g**), and dropout in (**h**).

We present some of the images augmented by the faster autoaugment algorithm in Figure 3. We observed that the resulting policy transforms images applying geometrical transformations such as horizontal flipping and shifting with mirroring. We also observed color transformations such as dropout, blurring, or changes in intensity. Notably, the algorithm learned that the problem was invariant to horizontal flipping and translations as well as to small changes in the pixel value such as blurring or dropout. Most importantly, it also learned to exclude vertical symmetry; vertical flipping did not form part of the learned policy.

**Figure 4:**
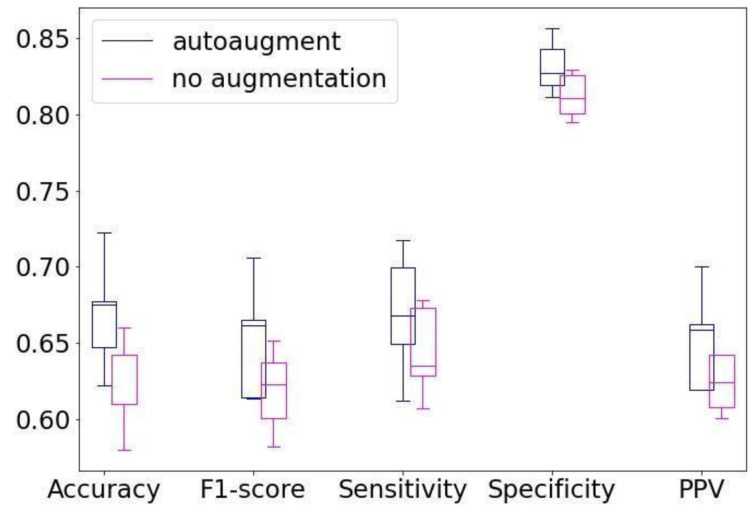
Cross-validation metrics for models trained with and without automated augmentation. The lower bar from the boxes above represents the minimum value, the lower bar of the box the first quartile, the bar in the middle the median, the upper box bar the third quartile, and the upper bar the maximum value. Models trained with augmentations performed significantly better than the model trained with no augmentations (*p* < 0.05).

**Figure 5:**
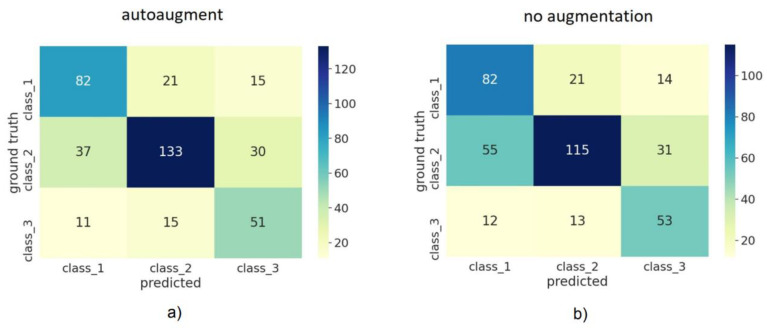
Average confusion matrix for cross-validation using automatic Image Augmentation in (**a**) and without augmentation in (**b**). It can be observed that using automated augmentation reduced the number of misclassifications for class 2 and increased the number of correctly classified images for this class. Confidence intervals were omitted for readability.

**Figure 6:**
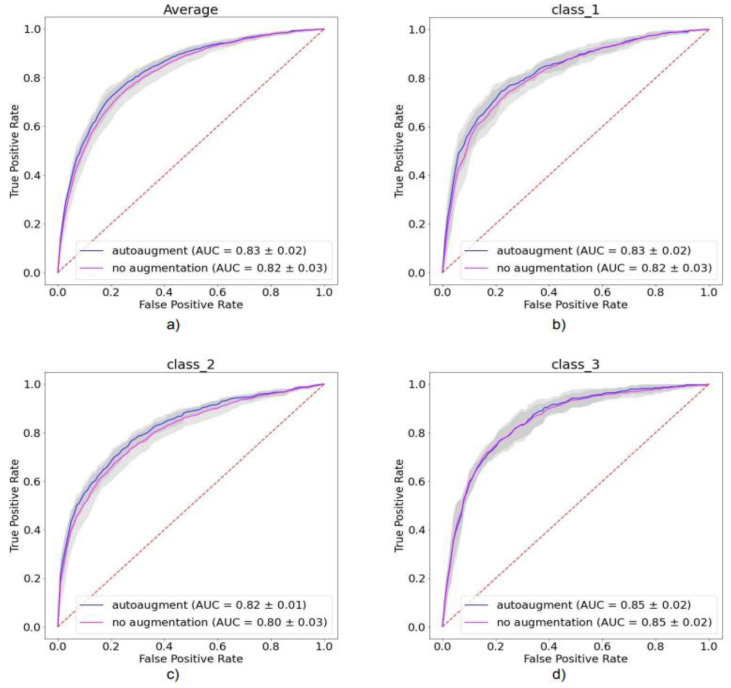
Average and class-wise receiver operating characteristic curves and area under the curve values for (**a**) average of all classes, (**b**) Angle class I, (**c**) Angle class II, and (**d**) Angle class III.

The impact of automated augmentation on accuracy estimates is displayed in Figure 4. We observed that the model trained with automatic augmentation performed generally better for all metrics than the model trained without augmentations; all metrics were significantly higher for augmented than non-augmented models (*p* < 0.05). We further compared how the models performed for each Angle class using the confusion matrix (Figure 5). For automated augmentation, we observed a significant increase in the number of correctly classified images from class 2 and a significant decrease in the number of misclassified images from the same class. Figure 6 shows the average and class-wise ROC and AUC values for augmented and non-augmented models.

### 3.3. Explainability

**Figure 7:**
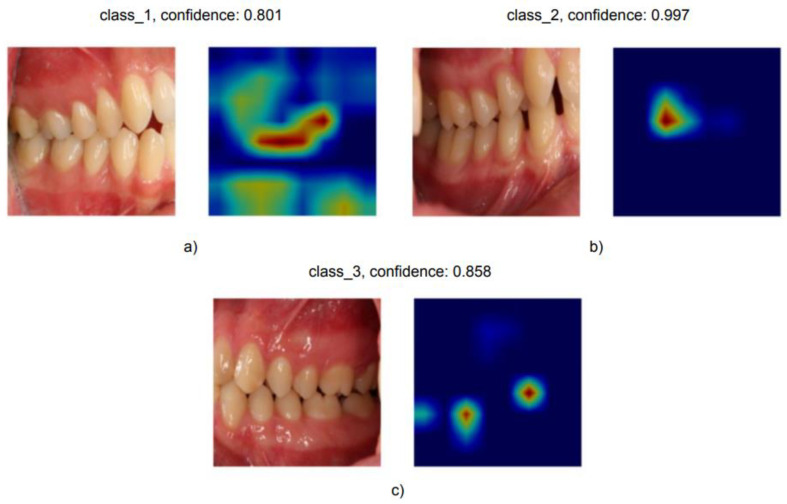
GradCAM visualization for some correctly classified images from Angle class I (**a**), class II (**b**) and class III (**c**). The red area indicates a high relevance for the particular outcome of the classification. Blue areas are not considered relevant for the Angle classification.

Finally, we were interested in interpreting the output of the models. Figure 7 shows the interpretability maps produced by GradCAM for images from one of the test splits. The red areas are the most relevant for the output category. As shown in these examples, it was noticeable that the model paid attention almost exclusively to areas relevant for determining the Angle class: the upper and lower first molars and, specifically, the relationship between the mesiobuccal cusp of the upper first molar and the groove between the mesial and middle cusps of the lower first molar.

## 4. Discussion

The aim of this study was to evaluate the effect of hyperparameter tuning and automatic image augmentation on a DL-based classification of the dental sagittal occlusion on intraoral photos. We had hypothesized that both strategies would improve the classification, and we accept that hypothesis. 

Currently, the Angle classification is recorded during clinical examination by an orthodontist by looking at the lower first molars and evaluating its sagittal relationship to the upper first molars. This evaluation is regularly trained in orthodontic courses during the dental education and needs knowledge, exercise, and suggestive experience in case the cusps are worn already at the time of the evaluation. Notably, classification results are highly dependent on the angle at which the practitioner is looking at the teeth (a perpendicular angle onto the buccal surfaces is ideal), which, in turn, depends on the patient’s mouth opening. 

Intraoral photo documentation is an additional part of the standard evaluation, and AI-based classification of the Angle class on photos may assist the clinical evaluation. Moreover, in the hands of less-experienced practitioners (postgraduate students), it may help in achieving high classification accuracy, allowing for targeted referral. AI-based systems may also be used for orthodontic training and patient communication. Furthermore, patients may be able to acquire lateral photos with their smart devices and to pre-check their Angle class, helping them to decide if there is a need to present themselves to an orthodontist for a further consultation or not. Moreover, photos and AI-based evaluation may be useful in monitoring orthodontic therapy by professionals.

Based on our study, a number of findings emerged.

First, hyperparameter tuning and automatic image augmentation are well-known techniques used to improve the performance of computer vision models. Using these techniques requires little technical effort and no domain-specific knowledge; a developer with no domain expertise can nevertheless significantly improve the performance of AI models for a particular field. This is particularly powerful in dentistry and medicine in general due to the high costs of training practitioners to develop this type of automatic diagnostic systems. The techniques used in this study offer the possibility of improving the diagnostic performance of models with minimal effort, which ultimately could translate into a better service and patient care.

Second, from the hyperparameter tuning experiments, we conclude that a combination of medium-sized learning rates and small batch sizes yielded the best results for accuracy and sensitivity. We also observed that the smaller the batch size, the higher the learning rate can be without a significant drop in performance. The learning rate had a larger effect on performance than batch size. Overall, the choice of the two hyperparameters considered in this study has a drastic impact, from useful accuracies at around 0.65 in the best-performing models to accuracies near zero for the worst ones. Further research is needed to confirm if the observed effects of hyperparameter tuning can be generalized to other DL tasks in dentistry, while modelers in the field should appreciate the relevance of this step for dental DL.

Third, automatic augmentation proved to have a positive influence on the performance of the models. The learned augmentation policy had a significant effect on all metrics, increasing them by around 5–10 percent. This effect was smaller than that of hyperparameter tuning, though, but is in line with effects for other medical applications [12,13,14]. Notably, and assuring, the augmentation policy learned the symmetry of the problem and discarded vertical flipping as one of the transformations. Moreover, the main impact of using automatic augmentation was reducing misclassifications of class I into class II. We did not test its impact on the generalizability or robustness of the model to other imagery (e.g., from other populations or image characteristics), which is where augmentation may be even more relevant.

Fourth, we showed that the models learned to classify based on features relevant for humans when classifying Angle classes I, II, and III, too. The resulting explainability is relevant to gauge medical logic and increase trust as well as to scrutinize failure cases. While, in many cases, the model highlighted an area of interest for orthodontists, it was observed that, in some cases, the model focused on a non-relevant area for the classification, a sign that the model might have learned misleading patterns, also known as shortcut learning. 

This study has a number of strengths and limitations. First, hyperparameter tuning and augmentation are standard instruments in dental DL but are usually chosen on a non-informed basis; the present study is the first one to systematically assess their effects for computer vision in dentistry. Our study serves to display the relevance of both factors—hyperparameter tuning having been found far more relevant than augmentation—and to inform modelers about potential choices to make. For example, balancing the learning rate and the batch size seems a useful approach, as the learning rate accounts for the magnitude of the update of the gradient for optimization, and the batch size indicates the number of samples that are optimized at once. A good combination of these two parameters stabilizes the optimization and can allow the model to reach lower values of the loss function and therefore increase performance. Second, and as a limitation, the used dataset was limited in its size and representativeness. As a consequence, the trained models showed only moderate accuracy and presumably limited generalizability. We accepted this caveat, as we aimed to test the effects of tuning and augmentation and not to train clinically useful models. Similarly, the employed photos were of high quality; if photos were of poor quality (e.g., not taken at a nearly 90° angle or show blurring, dropouts, etc.), this may impact on model accuracy, too. For training models fit for “real-world” application, a compromise of image quality may need to be accepted and such compromised images intentionally sampled into the dataset. Third, we explored the two modeling aspects in a controlled and separated fashion; joint variance would be of interest, too, and should be explored in future studies. Similarly, quantifying efficiency aspects when varying hyperparameter tuning and augmentation policy should be assessed. 

## 5. Conclusions

Computer vision models with small datasets can be sensitive to the choice of hyperparameters, particularly batch size and learning rate, as demonstrated in our experiments. Efficient hyperparameter tuning helped to identify the optimal values for maximizing the performance of the models and to avoid the overheads of a brute force or a manual search for optimal hyperparameter parametrization. Similarly, it is often a time-consuming task to design a good augmentation policy manually, and any prior information about symmetries of the data, etc. might not be available for the computer vision engineer but remains relevant for modeling. Automated augmentation can optimize the augmentation policy for a given problem and was shown applicable to a dental task in the present study.

## Data Availability

The data presented in this study are available on request from the corresponding author. The data are not publicly available due to data protection reasons.
